# Creating and Implementing a Community-Focused, Culturally Tailored Health Marketing Campaign to Address Menthol Cigarette Use in Los Angeles County

**DOI:** 10.5888/pcd21.230282

**Published:** 2024-04-18

**Authors:** Rachel Humphrey, Amy Truong, Renee Fraser, Tonya Gorham Gallow, Lori Fischbach, Tony Kuo

**Affiliations:** 1 Communications and Community Relations, Division of Chronic Disease and Injury Prevention, Los Angeles County Department of Public Health, Los Angeles, California; 2 Fraser Communications, Los Angeles, California; 3 Tobacco Control and Prevention Program, Division of Chronic Disease and Injury Prevention, Los Angeles County Department of Public Health, Los Angeles, California; 4 Research and Evaluation, Division of Chronic Disease and Injury Prevention, Los Angeles County Department of Public Health, Los Angeles, California; 5 Department of Family Medicine, David Geffen School of Medicine at University of California, Los Angeles (UCLA), Los Angeles, California; 6 Department of Epidemiology, UCLA Fielding School of Public Health, Los Angeles, California; 7 Population Health Program, UCLA Clinical and Translational Science Institute, Los Angeles, California

## Abstract

**Introduction:**

Menthol tobacco products have been marketed disproportionately to communities of color for decades.

**Methods:**

In Los Angeles County, California, a health marketing campaign, which used glossy visuals and attractive people in appealing poses, reminiscent of tobacco marketing tactics, was created and implemented to educate smokers on the health risks of using menthol cigarettes. The campaign encouraged smokers to make a quit attempt by offering access to free or low-cost resources through the *Kick It California* quitline and the *LAQuits* website (laquits.com). A survey tailored for public health professionals and community members from the approximately 382,000 people in the county who smoked menthol cigarettes and were exposed to their smoke (our primary audience) was administered to generate insights about this problem. Survey data were used to finesse the campaign creative materials prior to launch. Advertisement exposures, website visits, and quitline call volume were monitored and tabulated to assess the performance of the campaign.

**Results:**

At the conclusion of its initial run (February–April 2021), the “Done with Menthol” campaign had garnered more than 66 million impressions, received approximately 56,000 clicks on its various digital media platforms, and had click-through rates that surpassed industry benchmarks. The quitline call volume for African American and Latino subgroups were 1.9 and 1.8 times higher than the average inbound call volume for corresponding months during 2018 and 2019, respectively. In its second run (May–June 2023), the campaign garnered approximately 11 million additional impressions.

**Conclusions:**

Despite having a lower budget and fewer resources than the tobacco industry, the “Done with Menthol” campaign attained excellent reach and offered free, low-cost, and accessible resources to county residents interested in tobacco use cessation.

**Summary**

**What is known on this topic?**

Public health counter-advertising is a critical tool for combating disproportionate marketing of menthol tobacco products to communities of color.

**What is added by this report?**

This project describes how a local health department used appealing creative materials and messaging, reminiscent of tobacco marketing tactics, to address menthol cigarette use in Los Angeles County.

**What are the implications for public health practice?**

The “Done with Menthol” campaign resulted in more than 66 million impressions, and it referred many smokers to the state’s quitline and connected them to free and low-cost resources on the *LAQuits* website.

## Introduction

Vulnerable populations, especially communities of color, have long been a source of addiction and revenue for the tobacco industry, contributing significantly to tobacco-related illness and death rates in the United States (1,2). For example, 1 in 5 deaths every year in the US is attributed to tobacco use (3). Beginning in the 1960s, the tobacco industry has marketed menthol tobacco products to African Americans, often across several generations (2,4,5). To grow a new customer base, the industry has employed similar marketing tactics to entice Latino and LGBTQ+ groups (6,7). By appealing to customers using attractive models that look like them (eg, Black models) and building an image that amplifies the desire to assimilate into the “American” culture — placing disproportionate value on expensive clothes, sports cars, clubs, and music scenes and being young, fit, and happy — the tobacco industry has succeeded in encouraging people to try mentholated, flavored tobacco products (2,5).

In 2019, 85% of African Americans in the US who smoked used menthol cigarettes (8). In Los Angeles County, the prevalence was lower (13% of 382,000 people, or nearly 50,000 African American adults), but it was still higher than among Los Angeles County White smokers (3%) (9). The overall prevalence of tobacco use in the county is approximately 11% (893,000 smokers). Among people who received cessation services from the quitline, approximately 26.9% quit for 30 days and 13.9% quit for 6 months (10).

## Purpose and Objectives

To address local disparities in menthol cigarette use and to support a recently adopted flavor ban in Los Angeles County, the Tobacco Control and Prevention Program (TCPP) in the Los Angeles County Department of Public Health developed a health marketing campaign using health communications principles (11–13) and best practices from the literature for working with vulnerable populations (14,15). The campaign used many of the same marketing tactics as those used by the tobacco industry to convey the truth about menthol cigarette use: that their use can kill. A secondary campaign objective was to encourage quit attempts among smokers by referring them to free or low-cost cessation resources in the community.

## Intervention Approach

### Campaign development

To create a campaign that would resonate with its intended audience(s), TCPP contracted Fraser Communications (hereinafter, Fraser) to manage the campaign, including its development, production, media planning, and implementation. In collaboration with an African American marketing firm, Fraser set out to develop advertisements (ads) that mirror tobacco industry tactics, using them as familiar paths to delivering appealing, culturally appropriate cessation messaging.

To test whether these ads (eg, visual materials and text copies) would resonate with the intended audience, an online survey containing a series of agreement statements and open-ended questions was developed and administered to 2 groups of community members: 1) public health professionals, and 2) people who have had or currently have a flavored tobacco (eg, menthol) nicotine use disorder (NUD) or are exposed to menthol cigarette smoke (ie, current or prior smoker, or lives with someone who has a NUD (hereinafter, NUD respondents). The survey was conducted by a research and evaluation firm hired by Fraser. Administration of the English language survey was preceded by having each person in each of the groups review 4 social media ads and listen to two 30-second radio ads. Criteria for participation for the first group was that the prospective participant must be aged at least 18 years and work in the field of public health; participation criteria for the second group was that the prospective participant must be aged at least 18 years and have a NUD or live with a current smoker of menthol cigarettes.

Public health professionals were asked, based on their experiences, how people with a menthol-related NUD might react to the ads. The NUD respondents were asked about their reactions to the ads and opinions about the content. All surveys were completed during November and December 2020. The final enrollment numbers by group were 15 public health professionals and 27 NUD respondents or those exposed to menthol cigarette smoke. Not all members from each group fielded a complete survey.

### Campaign dissemination

**Figure 1:**
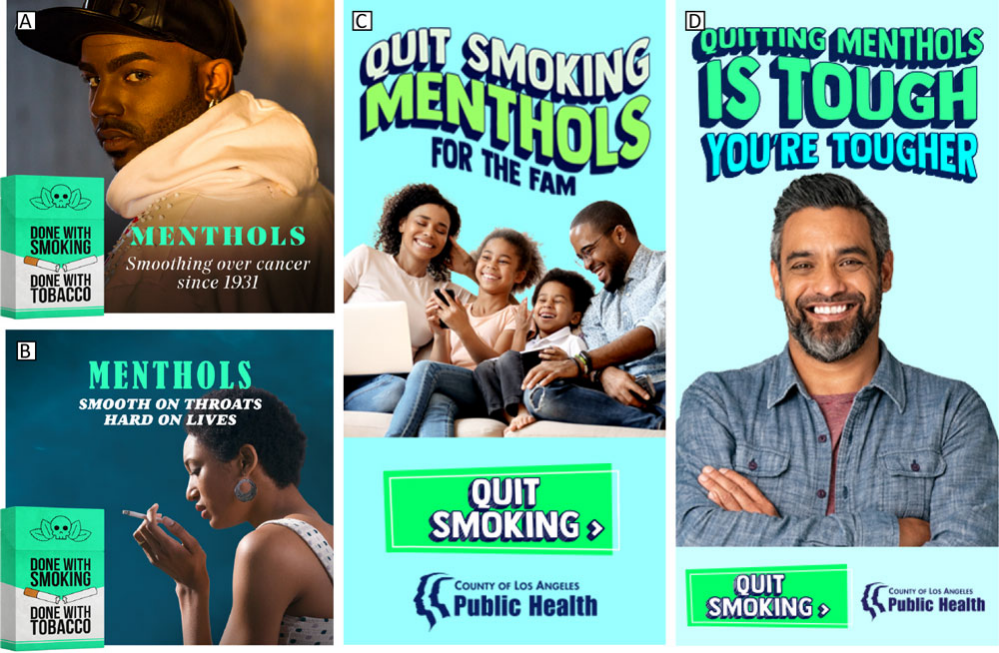
Los Angeles County Department of Public Health’s health marketing campaign, “Done with Menthol.” Photo A, “Menthols — Smoothing Over Cancer Since 1931”; photo B, “Menthols — Smooth on Throats Hard on Lives”; photo C, “Quit Smoking Menthols for the Fam”; and photo D, “Quitting Menthols is Tough, You’re Tougher.”

**Figure 2:**
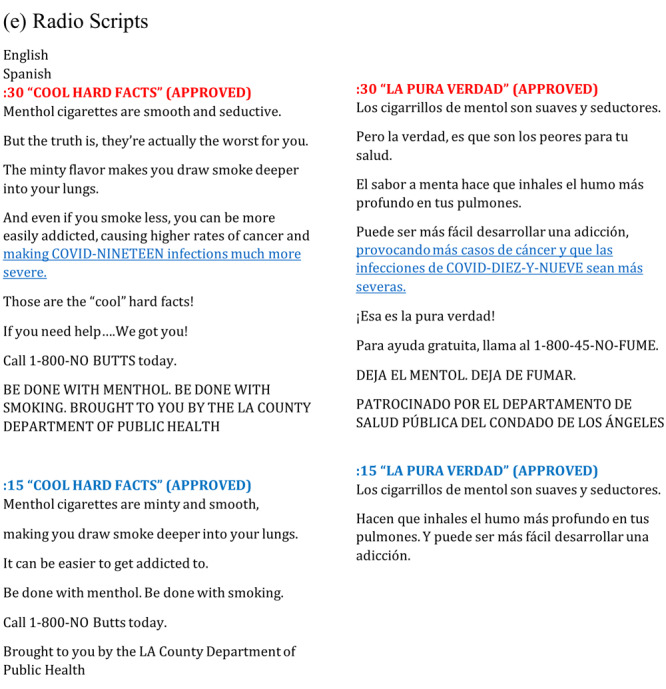
Los Angeles County Department of Public Health’s health marketing campaign, “Done with Menthol,” radio station advertisements with custom elements for station disc jockeys.

Guided in part by the transtheoretical model (Stages of Change framework) (16), the “Done with Menthol” campaign was disseminated from February 15 through April 4, 2021, and from May 8 through June 8, 2023. The ads were delivered in both English and Spanish; they ran on radio, out-of-home ads, in print (African American weekly and Spanish daily newspapers), on streaming radio, via social media (eg, Facebook, Instagram), and through targeted digital networks such as B Code and H Code (ie, digital pipelines for brands to connect with African American and Hispanic audiences, respectively). The campaign also ran through a partnership with Blavity, a Black-owned media technology company that focuses on Black culture via their online platforms. In its initial run, “Done with Menthol” promoted the negative health effects of menthol cigarette use and encouraged intended audiences to quit: “Menthols — Smoothing Over Cancer Since 1931” and “Menthols — Smooth on Throats Hard on Lives” (Figure 1). In the 2023 run, the messaging focused on self-motivation, promoting celebration of each quit attempt, and encouraging action by appealing to a person’s sense of family obligation and self-efficacy: “Quit Smoking Menthols for the Fam” and “Quitting Menthols is Tough, You’re Tougher” (Figure 1). The campaign promoted free and low-cost cessation resources (eg, a referral number to *Kick It California*, the state’s quitline) (17) and a call to action (ie, “learn more”) on the *LAQuits* website. Strategic media placements were key considerations in disseminating the campaign. For example, radio station ads included custom elements to authentically speak to specific audiences by way of radio station disc jockeys who are trusted community voices (Figure 2). Out-of-home ads were placed at convenience or liquor stores where people think of smoking or purchasing tobacco products.

## Evaluation Approach

Two data sources were used to assess reach and performance of the campaign: 1) standard metrics used by the marketing industry (18) to document media performance, which include the number of impressions, clicks, and click-through rates (CTRs); and 2) changes in quitline activities (eg, an increase in call volume during the campaign’s live runs, compared with corresponding months from the previous year). Although other measures of performance such as postcampaign surveys would have provided additional verification and insights into the ads’ effects, funding constraints restricted evaluation to the use of standard media metrics only.

For standard media metrics, “impressions” represent any audience interaction with a piece of content; they indicate the number of times intended audiences saw or heard the content. “Clicks” are the absolute counts of interactions with webpages, social media platforms, and other digital interfaces. CTRs indicate how often audiences click a link in one of the ads to access additional content. Collectively, these metrics provide a surrogate measure of overall reach and engagement by the campaign. All of the metrics’ data were managed and analyzed using standard software available through the public health department: Excel (Microsoft Corp) and SAS version 9.4 (SAS Institute, Inc).

The approach taken to document the campaign’s reach and performance did not allow for definitive identification of audiences who were from communities of color. However, ads were placed with media companies known for their large following of African American and Latino listeners or site users and for content tailored to the preferences of these groups. The high volume and frequency of the ads also helped ensure that exposure to these ads among these groups was substantive. For example, radio ads across 12 stations (including Spanish language) ran 125 times each week for about 5 weeks during the time the campaign was live.

## Results

### Formative work on creative materials

In the formative stage of campaign development, most of the public health professionals (n = 14; mean age, 51 y [range, 18–65 y]; 85.7% female) and NUD respondents (n = 23; mean age, 33 y [range, 20–46 y]; 73.9% female) who saw the ads reacted favorably to the creative content and design. For example, nearly 35% of NUD respondents agreed that the ads made them think about reducing their smoking in the future and about quitting tobacco use altogether (or never starting again). Twelve of the public health professionals (86%) believed the ads would be somewhat effective or very effective for audiences who had a NUD. Most survey participants, particularly the smokers, believed the ads were important, clear, told or taught them something new, and were worth sharing with friends or family members. More than half of professionals (64%) and NUD respondents (52%) agreed that the ads increased their concern about the risks of menthol cigarette use and their awareness of the state’s quitline; they also thought the information was credible because it came from a trustworthy source (ie, public health).

### Performance of the campaign

Although no one ad significantly outperformed the other, the collective reach and performance of the campaign were robust (approximately 382,000 people used menthol cigarettes in Los Angeles County at the time of the campaign) (9). In its initial run, the “Done with Menthol” campaign garnered more than 66 million impressions, received approximately 56,000 clicks on its various digital and social media platforms, and had CTRs that surpassed industry benchmarks (Table). In its second run (May–June 2023), the campaign garnered approximately 11 million more impressions. Ad performances by language and by medium varied, which is reflected by the campaign’s strategic ad placements in different geographic as well as specialty platforms or networks. For instance, in platforms like B Code where ads were tailored to the English-speaking African American and Black audience, English ads performed 24% better than Spanish ads.

**Table:** Performance of the “Done with Menthol” Campaign in Los Angeles County, February–April, 2021, and May–June, 2023

Campaign	Timeline	Objective	Intended audience	Media type	Impressions	Clicks	CTR^a^, %
*Done with Menthol*: “Smoothing Over Cancer Since 1931” and “Smooth on Throats, Hard on Lives”	First run: February 15, 2021, through April 4, 2021	To bring attention to the dangers of menthol cigarettes and encourage people to stop smoking, with an overall focus on reducing the use of menthol-flavored products in communities of color	People who smoke menthol cigarettes; Black and Latino men; aged 25–54 years. Radio demographic; English and Spanish language	Radio^b^ (with added value of >244 spots that ran as bonus)	16,443,402	—	—
				Streaming audio	1,198,943	646	0.05
				Programmatic banners	14,181,982	22,391	0.16
				Facebook/Instagram	2,610,795	22,678	0.87
				HCode Media (Latino/x)	1,012,336	7,994	0.79
				iOne (African American)	982,759	1,109	0.11
				Blavity	774,862	748	~0.07
				Outdoor messaging via 437 units: 128 bus tails, 8 posters, 4 bus shelters, 172 convenience store posters, and 125 digital POS screens at convenience stores	29,100,000	—	—
				Print: 2 ads in *LA Sentinel* and *Our Weekly* (Los Angeles, Antelope Valley) and 4 ads in *La Opinion* (Spanish)	400,546	—	—
				**Total**	**66,705,625**	**55,566**	—
*Done with Menthol* Campaign, “Quit Smoking Menthols for the Fam” and “Quitting Menthols is Tough, You’re Tougher”	Second run: May 8, 2023, through June 8, 2023	To bring attention to the dangers of menthol cigarettes and encourage people to stop smoking, with an overall focus on reducing the use of menthol-flavored products in communities of color	People who smoke menthol cigarettes; Black and Latino men; aged 25–54 years; Radio demographic; English and Spanish language	Programmatic banners	917,883	908	0.10
				Facebook/Instagram	754,708	12,840	1.70
				HCode Media (Latino/x)	1,181,613	1,030	0.09
				BCode Media (African American)	1,542,871	757	0.05
				Blavity	760,029	535	0.07
				Outdoor messaging via 59 units: 54 convenience store posters, 5 City Lights (coverage of West Hollywood, added coverage of San Fernando Valley)	6,300,000	—	—
				**Total**	**11,457,104**	**16,070**	—
*LAQuits.com*	February 1, 2021, through April 30, 2021	Website: resources for quit smoking tips, free or low-cost cessation support and link to *Kick It California* quitline	People who smoke menthol cigarettes; Black and Latino men; aged 25–54 years; Radio demographic; English and Spanish language	Website statistics and data tracking data via Web Analytics	Of the 66,000 website visit sessions and 56,000 new users during the campaign flight, it was estimated that at least 48,420 sessions and 40,484 new users were from the “Done with Menthol” campaign	—	—

Abbreviations: — , not applicable; CTR, click-through rate; POS, point of sale.

a For this campaign, CTR performances of key components were generally above industry benchmarks (ie, they exceeded expectations).

b Radio stations were selected to best reach both general market listeners and specific target audience(s). Many of them were ethnic media and language-specific news outlets (not an exhaustive list): KJLH-FM, KPWR-FM, KRRL-FM, KTWV-FM, and KDAY-FM, mostly Black, urban listeners; KRTH-FM, mostly English-speaking Hispanic and general market listeners; KLAX-FM, KWKW-AM (sports), KXOL-FM, and KSCA-FM, mostly Spanish-speaking listeners; KCEL-FM (Spanish), KUTY-AM, KGMX-FM, and KQAV-FM, listeners in the Antelope Valley (rural) area.

For *LAQuits.com*, website visits were mostly from mobile devices (78%), while almost 16% came from desktop or laptop computers and the remaining 6% from tablets. Length of time during website visits showed interest and time spent looking at webpage content, searching for more information. After visiting the “Menthol” English or Spanish landing webpage, users visited the *LAQuits.com* homepage and the “Stressed” webpage the most. Also, despite 2 other tobacco prevention campaigns (quit vaping and cessation) running concurrently in Los Angeles County, at least 48,420 and 40,484 of the total 66,000 web sessions and 56,000 new visitors, respectively, were attributed to the “Done with Menthol” campaign.

**Figure 3:**
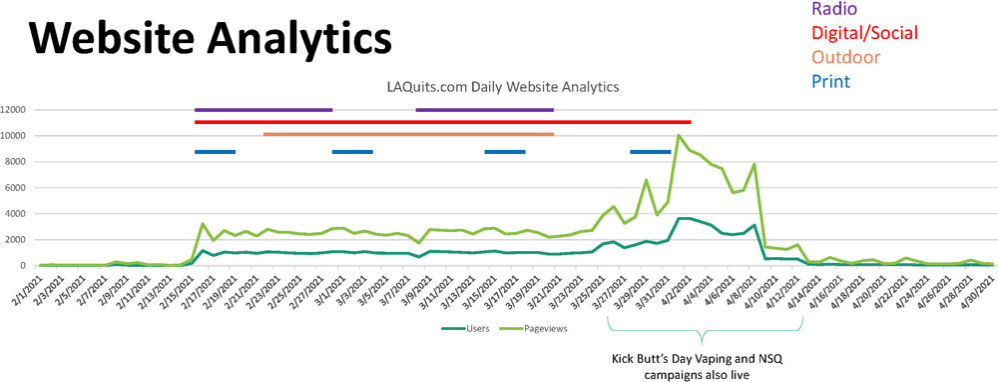
Los Angeles County Department of Public Health’s health marketing campaign, “Done with Menthol,” website analytics.

During the campaign’s initial run, the state quitline call volume for African American and Latino subgroups was 1.9 and 1.8 times higher than the average inbound call volume for the corresponding months during 2018 and 2019, respectively (Figure 3).

## Implications for Public Health

The “Done with Menthol” campaign used similar stylistic tobacco industry marketing tactics that drew people in with attractive human models, images, and slogans. At the same time, its messaging and visual materials motivated people to learn more about how to quit, especially when stress is a trigger for smoking. The campaign encouraged smokers to make a quit attempt with the aid of in-person and online resources such as the *Kick It California* quitline and the *LAQuits* website. The quitline is an effective venue for cessation because it provides people with the opportunity to speak to a live person (eg, an experienced counselor) in real time to obtain help with quitting tobacco. In Los Angeles County, this campaign was able to address many of the disparities it set out to improve, including strengthening public support for the County flavor ban and providing cessation resources to African American and other communities of color, and to LGBTQ+ groups.

The “Done with Menthol” campaign took advantage of the tobacco industry’s marketing strategies and leveraged them to increase interest in cessation, primarily through public awareness and referrals to free or low-cost in-person and online treatment resources. For local jurisdictions interested in reducing menthol cigarette use in their communities, the Los Angeles County experience could serve as a model of practice for how health marketing could be used to strategically combat this public health problem (11,12,19). The campaign could prove effective even in situations where the industry outspends local health departments in marketing and advertising dollars; for example, in 2019, the 4 major US cigarette companies spent more than $7.6 billion on advertising and promotion (20).

### Limitations

Although this project provides insights into a model of practice for developing and implementing a tobacco counter-advertising health marketing campaign, its performance evaluation has limitations. First, the formative stage of campaign development, although thorough in its approach to testing ad concepts, relied heavily on reactions and opinions from a small group of individuals, suggesting that selection and social desirability biases may have affected their responses. Second, even after coordinating with the California Tobacco Control Program on the timing of their tobacco counter-advertising, many state-sponsored ads were still running in the LA media market (ie, their impact on quitline call volume and *LAQuits* website visits could not be distinguished). Third, due to varying political affiliation, ideology, and support of certain policies in a given community, the “Done with Menthol” campaign, like many others before it, may not be generalizable to audiences in other US jurisdictions. Lastly, although the campaign used standard industry metrics to gauge media reach and engagement, these communications measures were not designed to demonstrate causation or show health impact. A more rigorous design, including the use of a pre-/post-campaign survey, would be required to investigate such effects. In this project, this kind of design was not possible because funding was lacking to support research or rigorous program evaluation.
